# Action Recognition and Movement Direction Discrimination Tasks Are Associated with Different Adaptation Patterns

**DOI:** 10.3389/fnhum.2016.00056

**Published:** 2016-02-23

**Authors:** Stephan de la Rosa, Mina Ekramnia, Heinrich H. Bülthoff

**Affiliations:** Department of Perception, Cognition, and Action, Max Planck Institute for Biological CyberneticsTübingen, Germany

**Keywords:** action recognition, visual adaptation, action observation, movement direction, action adaptation, high-level adaptation, direction discrimination, action discrimination

## Abstract

The ability to discriminate between different actions is essential for action recognition and social interactions. Surprisingly previous research has often probed action recognition mechanisms with tasks that did not require participants to discriminate between actions, e.g., left-right direction discrimination tasks. It is not known to what degree visual processes in direction discrimination tasks are also involved in the discrimination of actions, e.g., when telling apart a handshake from a high-five. Here, we examined whether action discrimination is influenced by movement direction and whether direction discrimination depends on the type of action. We used an action adaptation paradigm to target action and direction discrimination specific visual processes. In separate conditions participants visually adapted to forward and backward moving handshake and high-five actions. Participants subsequently categorized either the action or the movement direction of an ambiguous action. The results showed that direction discrimination adaptation effects were modulated by the type of action but action discrimination adaptation effects were unaffected by movement direction. These results suggest that action discrimination and direction categorization rely on partly different visual information. We propose that action discrimination tasks should be considered for the exploration of visual action recognition mechanisms.

## Introduction

Humans are social beings and recognizing what another person is doing and in which direction the action is executed are important pieces of information for successful social interactions. Here we examined to what degree visual processes used for making directional judgments about an action and for recognizing the type of action rely on similar visual information.

Much of our understanding about the neural architecture of action recognition (Giese and Poggio, 2003; Lange and Lappe, 2006; Fleischer et al., 2013; Theusner et al., 2014) comes from research that mainly relied on biological motion stimuli (Johansson, 1973) showing locomotive (e.g., walking; Kozlowski and Cutting, 1977, 1978; Mather and Murdoch, 1994; Casile and Giese, 2006; Jokisch et al., 2006) or object directed actions (e.g., knocking; Runeson and Frykholm, 1983; Sokolov et al., 2011) and combined these stimuli with left-right (Chang and Troje, 2009; Theusner et al., 2011; Vangeneugden et al., 2014) or forward-backward (Beintema et al., 2006; Lappe et al., 2015) direction discrimination tasks. The probing of action recognition with tasks that require participants to distinguish different actions has received much less attention (Dittrich, 1993; de la Rosa et al., 2014, 2015).

Physiological results obtained with macaque monkeys demonstrate a close link between movement direction and action selectivity. Neurons in STS that were sensitive to a particular action showed only sensitivity to a particular direction of an action (Oram and Perrett, 1996). Although it is unknown to what degree this activation correlates with the actual percept of an action, these results suggest a close relationship between movement direction and action type within single neurons in STS. In contrast, one could argue that the direction of an action only carries limited informational value about the type of executed action, i.e., whether the person is carrying out a handshake or a high-five. For example, the biological motion patterns of a handshake or a high-five are both associated with a forward directed arm motion. Hence, inferring the type of action based on movement direction alone (e.g., forward-backward movement) is difficult. An important open question is the extent to which visual information involved in the visual discrimination of action direction contributes to the discrimination to different actions (e.g., telling a handshake from a high-five).

Here we used an adaptation paradigm to examine the degree to which movement direction information contributes to action discrimination. In our adaptation experiment, participants view an action, e.g., a handshake or a high-five, for a prolonged amount of time (adaptation) and immediately afterwards report their perception of an ambiguous test stimulus that contains kinematic elements of both a handshake and a high-five by means of action morphing. Participants frequently report that the ambiguous test stimulus looks more like a waving if they had been previously adapted to a handshake. Likewise, they report to see a handshake if they had seen a waving prior to the presentation of the test stimulus (de la Rosa et al., 2014). Hence, the prolonged viewing of an action transiently alters the perception of a subsequent ambiguous action away from the adapted action (action adaptation). The effect can be experienced in Supplementary Video 1.

Adaptation effects allow a behavioral assessment of the tuning properties of visual processes (Webster, 2011), because adaptation is believed to change the response properties of the visual processes engaged in the perception of the adaptor. If these visual processes are also involved in the perception of the test stimulus, the alteration of the response properties during adaptation will also affect the perception of the test stimulus. The resulting perceptual change of the test stimulus is referred to as adaptation effect. Adaptation effects typically decrease with increasing dissimilarity between the adaptor and test stimulus. Hence, one can measure the magnitude of the adaptation effect as a function of adaptor-test similarity to examine the tuning properties of the underlying visual processes.

Here we used this logic to examine the degree to which action discrimination processes are sensitive to action movement direction (e.g., forward vs. backward movement). We reasoned that if action discrimination processes encode action in a movement direction specific way (e.g., Oram and Perrett, 1996), then adaptation effects should depend on movement direction in an action discrimination task. Likewise, we investigated the sensitivity of direction discrimination processes to action information (e.g., handshake vs. high-five action). If visual processes underlying direction discrimination encode movement direction in an action specific way, then adaptation effects should depend on the action in a direction discrimination task.

**Figure 1:**
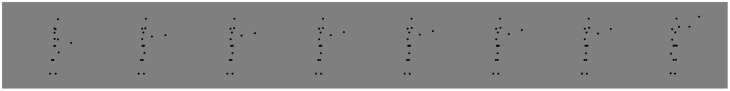
**Static images of the action morphs showing the peak of the action**. The morph values are (from left to right): 0 (handshake), 0.35, 0.383, 0.417, 0.45, 0.483, 0.517, 0.55, 1 (high-five). First and last stimuli served as adaptors (here only a snapshot is shown as they were presented as movies). The other stimuli served as test stimuli (these were presented as static images).

To this end, participants adapted to different movement directions and actions in four different conditions showing different action movies as adaptors: normal played handshake movie, reverse played handshake movie, normal played high-five movie, and reverse played high-five movie. Participants always saw the same set of test stimuli in each adaptor condition, namely briefly presented static images that were ambiguous with respect to the action type and movement direction (see Figure 1). In different experimental conditions participants judged either the perceived movement direction (direction discrimination task) or the action type (action discrimination task) of the test stimuli.

To generate test stimuli that were ambiguous with regards to their action type, we calculated the weighted linear combination of corresponding body points between two temporally aligned motion capture sequences. One motion capture sequence showed a handshake and the other a high-five action. We only showed the peak frame of the morphed action as a test stimulus. The peak frame was chosen from a recording in which an actor executed the action starting from a neutral pose (standing with arms aligned to the side of the body) and after execution returned to the neutral pose again. The peak frame was the frame immediately before the actor initiated the (backward) movement to go back into the neutral pose again. Because static images do not show any movement, test stimuli were ambiguous with respect to the movement direction. Moreover, they were also ambiguous with respect to the action category since they displayed body postures that contained elements of both a handshake and high-five action. For the direction discrimination task we asked participants to report whether the frame of the action was taken from a clip that was played forward or backward. For the action discrimination task we asked participants whether the action looked more like a handshake or a high-five.

## Experiment

### Methods

#### Participants

Previous research indicated that sample sizes of about 15 participants are sufficient to reveal adaptation effects (de la Rosa et al., 2014). We therefore recruited 15 participants (mean age 28.3 years; *SD* = 9.73; 12 females) from the local community in Tübingen. Participants were naïve with respect to the task and had normal or corrected-to-normal vision. Participants gave their written informed consent prior to the experiment. They received 8€/h as compensation for their participation. The experiment was conducted in accordance with the Declaration of Helsinki and was approved by the local ethics committee of the University of Tübingen.

#### Stimuli and apparatus

The stimuli (one person conducting a handshake and one person conducting a high-five) were taken from a motion capture stimulus set of a previous study (de la Rosa et al., 2013). Stimuli of this stimulus set contained the three-dimensional (3D) spatial coordinates of body joints unfolding over time. The handshake action was 1.21 s long and the high five action was 1.41 s long. For both actions the person started from an standing pose and then carried out the action with another person (who was not shown in the experiment). The action movie was cropped to the subjectively perceived peak of the action by the first author. The peak frame was defined as the frame immediately before the actor initiated the (backward) movement to go back into the neutral pose again. We created point light stimuli from the 3D models of the actions. The stimuli were presented on a DELL PC with a DELL Display running Windows 7, and an English keyboard layout. The display was a 24 inch display with a screen resolution of 1280 × 1024 pixels. We used a custom written MATLAB and Psychophysics Toolbox 3 software (Kleiner et al., 2007) for the stimulus presentation and response collection. All experimental stimuli were created by calculating the weighted average of each 3D joint position between the two actions for each animation frame separately. These points were then orthogonally projected to obtain a 2D movie frame. After the morph all stimuli (i.e., adaptors and tests) had a length of 1.21 s. Examples of the stimuli are shown in Figure 1.

#### Procedure

We followed a similar procedure to Barraclough and Jellema (2011). The experimental manipulations were tested in separate experimental blocks. An experimental block started with 30 adaptor presentations with an 200 ms adaptor inter stimulus interval (ISI) followed by 21 experimental trials. An experimental trial consisted of three presentations of an adaptor, followed by a 300 ms 1000 Hz tone, and the presentation the 100 ms test stimulus. The adaptor-test ISI was 300 ms. The tone indicated the onset of the test stimulus. The test stimulus probed participants' performance at one of the 7 morph values (0.35, 0.383, 0.417, 0.45, 0.483, 0.517, 0.55), which resulted in an ambiguous action percept by most of the participants in a pilot experiment. Participants were told that the briefly presented test stimulus was taken from a movie sequence either showing a person making a forward or making a backward action movement. Each of the 7 test stimuli was shown three times for a total of 21 trials per experimental condition. We calculated the high-five or the forward response proportion (depending on the task) from these 21 trials. In separate experimental blocks participants answered the questions “Was the image taken from a forward (z) or backward (‘.’) playing movie?” (direction discrimination task) and “Did the action look more like a handshake (z) or high-five (‘.’) ?” (action discrimination task). The letters in the brackets corresponded to the response keys associated with the corresponding action answers on an English keyboard layout. The assignment of answer options and response keys was counterbalanced across participants. Participants' responses were not time restricted. Point light actors and all text were presented in the center of the screen in black on a mid level gray background. There was a total of 8 experimental blocks: 2 discrimination tasks × 2 movement directions × 2 adaptors. The testing order of the experimental blocks was randomized across participants. At the very beginning of the experiment we always measured action categorization and direction discrimination of the test stimuli without the presentation of adaptors (baseline). That is, we had two baseline conditions - one for the action categorization and one for the direction discrimination task (testing order counterbalanced across participants). Each baseline probed the 7 morph levels three times (for a total of 21 trials) in random order. A paired *t*-test comparing the response proportions of two-baseline conditions (i.e., forward responses in the direction discrimination task and high five responses in the action discrimination task) revealed a tendency for a difference that did not reach statistical significance, *t*_(14)_ = 2.06, *p* = 0.058, M_difference_ = 0.143, SD_difference_ = 0.268.

## Results

We calculated the proportion of high-five (action discrimintation task) or forward (direction discrimination task) responses from the 21 trials (=seven morph levels each shown three times) of each condition. For the action discrimination task, we subtracted the proportion of high-five responses in the experimental conditions from high-five response proportion of the baseline condition in order to measure the adaptation effect. Likewise, we subtracted the proportion of forward responses of the experimental conditions from the one of the baseline condition to measure the adaptation effect in the direction discrimination task.

We analyzed the effect of task (direction vs. action discrimination), movement direction (forward vs. backward), and action type (handshake vs. high-five) with a completely within subject linear mixed model with maximum likelihood estimations. Participants were treated as a random effect and task, movement direction, and action type as fixed effects. In line with the suggestion to use a maximal random effects structure (Barr et al., 2013) we allowed random intercept and slopes per participant for each of the three factors. The analysis showed a significant main effect of action type, $\chi_{\left(1\right)}^{2}=23.$95, *p* < 0.001, and task, $\chi_{\left(1\right)}^{2}=8.$84, *p* = 0.003. The interaction between task and action type was also significant, $\chi_{\left(1\right)}^{2}=30.$07, *p* < 0.001. Most importantly, the three way interaction between task, movement direction, and action type was significant, $\chi_{\left(1\right)}^{2}\;=\;3.95$, *p* = 0.047 suggesting that the interaction of action type and movement direction depended on the recognition task. We therefore present the results from the direction and the discrimination task separately.

### Direction discrimination task

**Figure 2:**
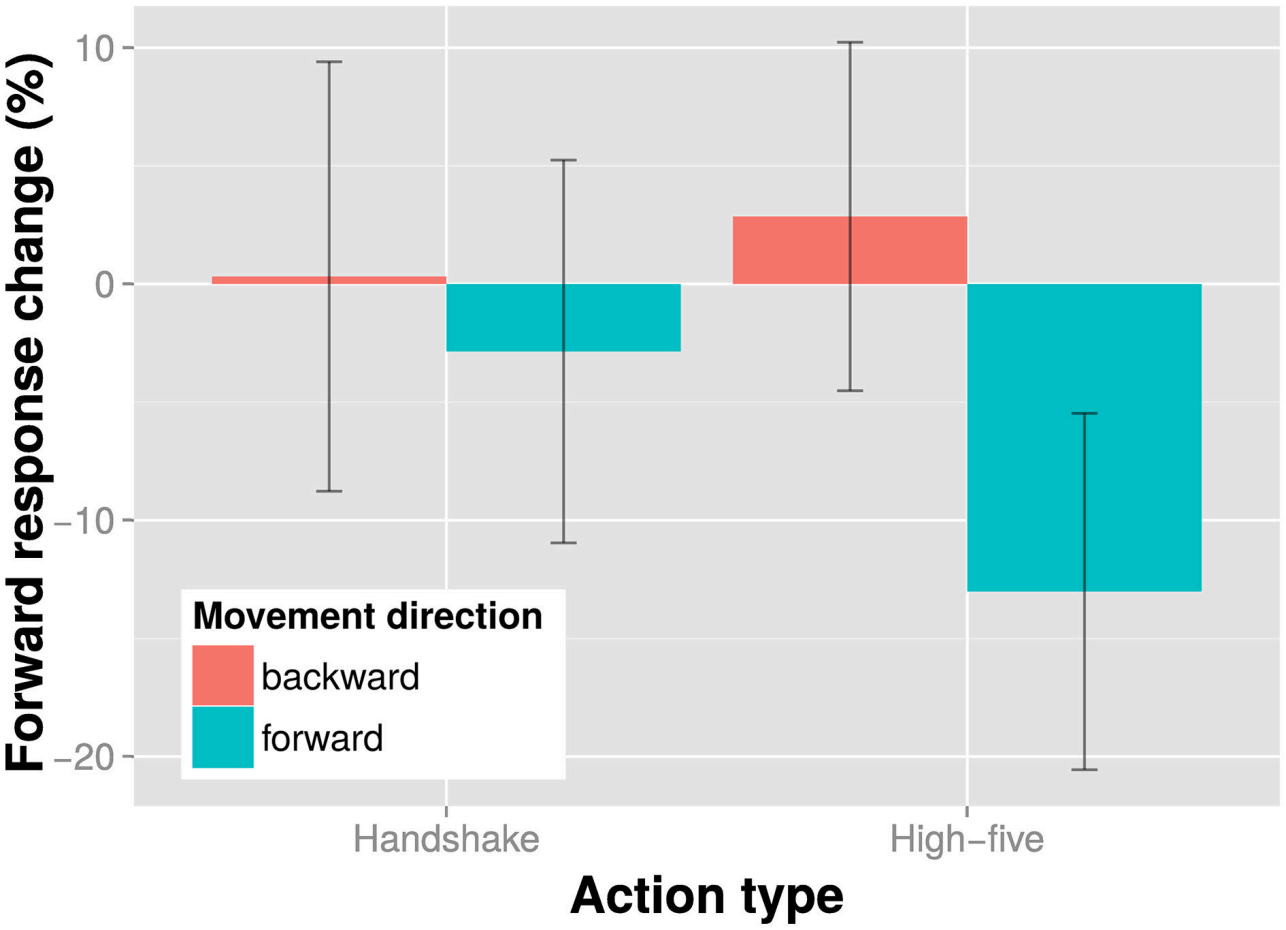
**Results of the direction discrimination task**. Shown is the percent response change of the “forward” responses in the experimental conditions relative to the baseline condition (no adaptor presentation) for each action type and movement direction adaptor separately. Bars indicate one SE from the mean.

The results of the direction discrimination task are shown in Figure 2. The figure suggests that forward and backward action adaptors induced a small adaptation effect with handshake actions and a larger adaptation effect with high-five actions: Seeing a forward moving action caused participants to perceive the test stimulus as more backward moving and vice versa. This antagonistic effect of the adaptor is in line with previous studies (e.g., Theusner et al., 2011) and demonstrates that it is possible to measure direction discrimination adaptation effects with static test images. We examined the effect of movement direction and action type on adaptation in a direction discrimination task in an all within subjects linear mixed model with a maximal random effects structure (per participants intercepts and slopes for action type and movement direction). We found no main effect of action type, $\chi_{\left(1\right)}^{2}=0.$23, *p* = 0.627, no main effect of movement direction, $\chi_{\left(1\right)}^{2}=0.$23, *p* = 0.629, but a significant interaction of action type and movement direction, $\chi_{\left(1\right)}^{2}=4.$98, *p* = 0.026. The significant interaction indicates that direction discrimination adaptation depends on the type of action. Hence, movement direction information does not seem to be encoded independent of action type.

### Action discrimination task

**Figure 3:**
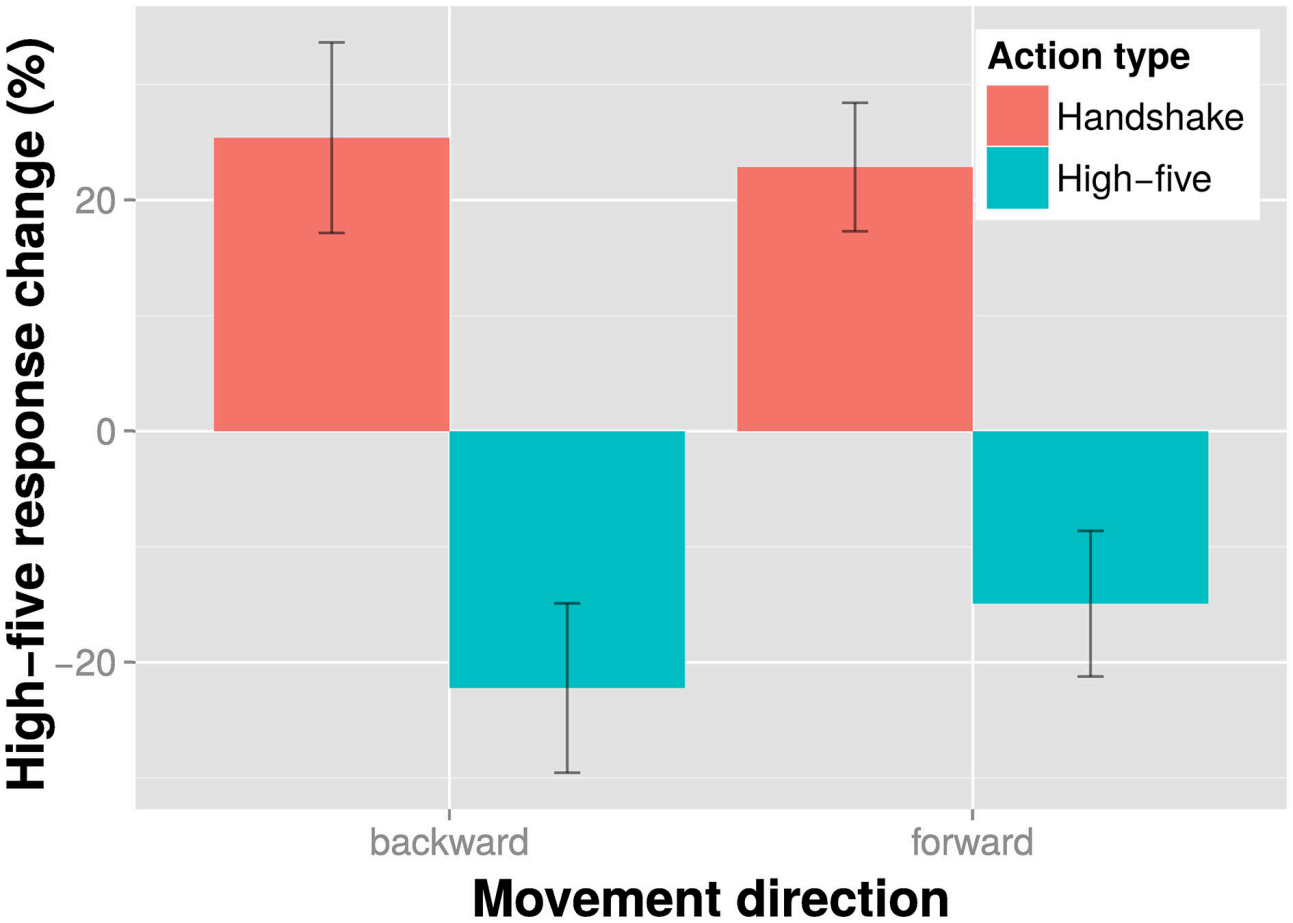
**Results of the action discrimination task**. Shown is the percent response change of the “high-five” responses in the experimental conditions relative to the baseline condition (no adaptor presentation) for each action type and movement direction adaptor separately. Bars indicate one SE from the mean.

The results of the action discrimination task are shown in Figure 3. The results indicate a clear antagonistic adaptation effect: when participants saw a high-five adaptor, they were more likely to perceive the ambiguous test as a handshake, and vice versa. Additionally, it seems that backward and forward movement had little effect on action discrimination adaptation effects. We examined the influence of action type and movement direction on adaptation in an action discrimination task in an all within subjects linear mixed model with a maximal random effects structure (per participants intercepts and slopes for action type and movement direction). We found a significant effect of action type, $\chi_{\left(1\right)}^{2}=23.$83, *p* < 0.001, indicating that action discrimination adaptation depended on the displayed action. However, the effect of movement direction on action discrimination was non-significant, $\chi_{\left(1\right)}^{2}=0.08$, *p* = 0.777. Likewise the interaction of movement direction and action type was also non-significant, $\chi_{\left(1\right)}^{2}=0.$63, *p* = 0.428. Overall, these results are in line with the suggestion that adaptation in action discrimination tasks is little affected by movement direction.

## Discussion

The purpose of the current study was to examine the degree to which direction discrimination depends on action information and action discrimination depends on movement direction information. Our results show that the magnitude of adaptation effects for direction discrimination depend on the type of action. Hence, visual mechanisms for recognizing the movement direction of an action seem to encode movement direction information in an action specific manner. In contrast, adaptation effects for action discrimination are relatively unaffected by movement direction. Because modulations of adaptation effects are informative about the tuning properties of the underlying visual properties (Webster, 2011), the results suggest that action discrimination processes encode actions independent of their movement direction in the current experiment. In contrast, direction discrimination processes seem to encode movement direction in an action specific manner in the present experiment.

To what degree do these results generalize to other actions? We would like to point out that action discrimination is likely to be only little effected by the movement direction of the action if movement direction is not an action defining feature. If movement direction is a defining feature for the action, possible interactions between movement direction and action recognition might occur. For example, Barraclough et al. (2009) showed that playing a movie of an object grasping action backwards resulted in the perception of a placing action. In their study, movement direction defined the type of action (i.e., placing vs. grasping action). Using these actions as adaptors, Barraclough and colleagues found adaptation effects in a weight discrimination task to be movement direction specific. It is possible that movement specific adaptation effects will also occur in an action discrimination task because grasping and placing actions in this example were identical except for the movement direction. Yet, we would like to point out that the same action carried out with a different action intention under real-life conditions is likely to result in different kinematic patterns, which are reliably picked up be the observer. For example, Georgiou and colleagues showed that grasping an object when cooperating and grasping the same object when competing with another person results in different hand movement patterns (Georgiou et al., 2007), which the observers used to infer to goal of the action (Sartori et al., 2011). Hence actions (e.g., grasping and placing) are likely to be associated with different kinematic patterns (other than movement direction), which might be used for action discrimination. In line with this idea other data in our lab shows that adaptation effects in action discrimination tasks reliably occur with actions that are associated with the same movement direction, where directional cues are little informative about the type of action, e.g., discriminating punch vs. fist-bump, catching vs. taking, throwing vs. giving. However, more research is needed to determine whether action discrimination of actions with clearly different movement directions rely solely on movement direction cues. As of now, we make the conservative suggestion that action discrimination is encoded independently of movement direction only as long as movement direction between the two actions is not clearly different.

Other evidence is in line with the idea that action discrimination adaptation effects are not merely driven by visual information about the movement direction. We have previously shown that adaptation effects in an action discrimination task are modulated by the immediate action context preceding an adaptor (de la Rosa et al., 2014). Specifically, the size of the adaptation effect was different depending on whether the adapting action was embedded into a friendly or hostile action context. This effect seem to be mainly caused by the context because the adapting and the test action stimuli were physically identical in both action context conditions. These findings suggest that at least part of the adaptation effect in action discrimination task is not based on visual action information (e.g., movement direction information). At the same time we found evidence for visual information being important for action discrimination adaptation. For example, in a control condition of de la Rosa et al. (2014) the action adaptor stimuli were replaced by action words. In this case, action discrimination adaptation effects disappeared. Overall, we suggests that visual action information alone (e.g., movement direction information) is not suffice to explain action adaptation effects. Instead, other visual processes (e.g., top-down influences from the action context) also influence action adaptation effects.

How can the present results be explained in terms of existing action recognition models (Giese and Poggio, 2003; Lange and Lappe, 2006; Fleischer et al., 2013)? We suggest that action specific direction discrimination adaptation effects can be well explained by adaptation of action specific motion units. In terms of existing models these units can be either the optic flow pattern neurons (Giese and Poggio, 2003) or the body posture specific motion templates on the second stage (Lange and Lappe, 2006). Because these units are action specific, adaptation to movement direction of one action does not necessarily influence movement direction judgments about another action. In contrast, action discrimination adaptation effects seem be owed to adaptation of visual processes located at a later processing stage where action representations are largely invariant to movement direction. Possible candidates are, for example, action-selective neurons (Fleischer et al., 2013).

Overall, the present results call for caution about generalizing results from forward-backward direction discrimination tasks to action discrimination tasks. It is likely that generalization from left-right discrimination tasks to action discrimination require similar careful consideration. Specifically, our findings suggests that direction discrimination task results are specific to the probed action and do not necessarily apply to other actions. At the same time, action discrimination processes of the two probed actions are much less sensitive to the movement direction of an action compared to the type of action. The visual processes underlying direction and action discrimination seem to rely to different amounts on action and on movement direction information and therefore seem to be associated with different response properties (at least for the actions probed in the present study).

It is important to note that our results are in line with the suggestion that action discrimination adaptation effects are based on motion information. The fact that action discrimination adaptation of the two probed actions is little affected by forward-backward playing of the actions only shows that the movement direction but not motion *per se* have little effect on action discrimination mechanisms. Furthermore, it is important to note that the non-significant effect of movement direction on action discrimination cannot be taken as evidence for movement direction not having an influence on action discrimination. Rather our results suggest that action information has a larger effect on action discrimination than movement information.

To what degree are action adaptation effects bound to social actions? For example, is it possible to observe adaptation effects also for object-directed actions, e.g., picking up an object or moving marbles in a jar. More recent evidence shows that adaptation effects are also observed for weight judgments (Barraclough et al., 2009) and directional judgments of object directed actions (Barchiesi et al., 2012). In this light it seems likely that the social nature of an action is not a prerequisite for adaptation effects to occur.

In conclusion, we demonstrate that adaptation effects in a direction discrimination task depend on the action type and therefore direction discrimination results cannot be necessarily generalized to other actions. Moreover, adaptation effects in action discrimination tasks are relatively little affected by movement direction. An open question remains whether this also holds for actions with very different movement directions. In sum the results suggests that action discrimination processes rely on different visual information than direction discrimination processes (at least for the actions assessed in the present study). We therefore advocate the use of so far less used but socially very relevant action discrimination tasks in the examination of action recognition processes.

## Author contributions

SDLR developed the study. ME collected the data, and SDLR analyzed the data. All authors contributed to the manuscript.

### Conflict of interest statement

The authors declare that the research was conducted in the absence of any commercial or financial relationships that could be construed as a potential conflict of interest.
